# Study of the Properties of Antifriction Rings under Severe Plastic Deformation

**DOI:** 10.3390/ma15072584

**Published:** 2022-03-31

**Authors:** Irina Volokitina, Alexandr Kolesnikov, Roman Fediuk, Sergey Klyuev, Linar Sabitov, Andrey Volokitin, Talgat Zhuniskaliyev, Bauyrzhan Kelamanov, Dauren Yessengaliev, Almas Yerzhanov, Olga Kolesnikova

**Affiliations:** 1 Department of Metallurgy and Mining, Rudny Industrial Institute, Rudny 111500, Kazakhstan; irinka.vav@mail.ru; 2 Department of “Life Safety and Environmental Protection”, M. Auezov South Kazakhstan University, Shymkent 160012, Kazakhstan; ogkolesnikova@yandex.kz; 3 Polytechnic Institute, Far Eastern Federal University, 690922 Vladivostok, Russia; 4 Peter the Great St. Petersburg Polytechnic University, 195251 St. Petersburg, Russia; 5 Belgorod State Technological University Named after V.G. Shukhov, 308012 Belgorod, Russia; klyuyev@yandex.ru; 6 Kazan Federal University, 420008 Kazan, Russia; sabitov-kgasu@mail.ru; 7 Department of Metal Forming, Department of Metallurgy and Materials Science, Karaganda Industrial University, Temirtau 101400, Kazakhstan; talgat.zhuniskaliev@mail.ru (T.Z.); kaf-omd@mail.ru (A.Y.); 8 Department of Metallurgy and Mining, K. Zhubanov Aktobe Regional University, Aktobe 030000, Kazakhstan; kelamanov-b@mail.ru (B.K.); dauralga@mail.ru (D.Y.)

**Keywords:** severe plastic deformation, antifriction rings, brass, microstructure, properties

## Abstract

The paper studies the properties of brass workpieces for antifriction rings under severe plastic deformation by high-pressure torsion. The evolution of microstructure and mechanical properties of deformed workpieces after six cycles of deformation by high-pressure torsion at 500 °C have been studied. All metallographic studies were performed using modern methods: transmission electron microscopy (TEM) and analysis electron back scatter diffraction patterns (EBSD). The deformation resulted in an ultrafine grained structure with a large number of large-angle boundaries. The strength properties of brass increased compared to the initial state almost by three times, the microhardness also increases by three times, i.e., increased from 820 MPa in the initial state to 2115 MPa after deformation. In this case, the greatest increase in strength properties occurs in the first two cycles of deformation.

## 1. Introduction

Antifriction materials are used to increase the durability of friction-exposed mechanisms and machine surfaces and to reduce friction losses. Antifriction rings are often used in technology because they interact with the support ring through an intermediate ring made of material with a higher tensile strength than the antifriction ring. Such rings are occasionally produced by casting. When cast, most metals and alloys have a coarse-grained structure (d > 100 µm) [1,2,3]. The following traditional methods are most commonly used in industry to refine the structure of such metals: heat treatment based on phase transformations, cold pressure treatment of metals followed by heat treatment, often in the form of recrystallization annealing, and thermomechanical treatment. Such conventional methods allow to grind the microstructure to ultrafine grains (d ≈ 1–10 μm) [4,5,6,7,8]. In recent years, many articles have been written showing that there are severe plastic deformation (SPD) methods that can remove this limitation and grind the microstructure to 0.1 μm and below directly during deformation [9,10,11,12,13,14,15].

Currently, there are a large number of SPD methods, the main ones being equal-channel angular pressing (ECAP) [16,17,18] and high-pressure torsion (HPT) [19,20,21,22], but, so far, they have only been used on a laboratory scale. In order to expand the scope of ultrafine and nanomaterials as structural, applicable for the manufacture of parts with unique physical and mechanical properties, it is necessary to create a method that allows the production of semi-finished products with enhanced properties. These properties include: high strength characteristics and homogeneous equiaxial ultrafine grain structure in both longitudinal and transverse sections; and high proportion of large-angle boundaries with no sharp texture. In addition, the new method must take into account plant requirements, is not difficult to adapt to the already available equipment, and has inexpensive and technological tooling. Such a method should be sought among the cyclic methods of metal forming. The most used and investigated of these methods are: screw extrusion, equal-channel angular pressing, and high-pressure torsion, which allow the deformation to maintain the original volume shape of the workpiece, regardless of the degree of deformation applied to it.

Because of its ability to strongly deform the sample (with a true strain of ≥ 10) [23] and to achieve highly refined grains with exceptional strength [24], the HPT method, which involves treating the sample by deformation under pressure and compression torsion, has shown great importance and become one of the main SPD methods [25,26]. However, although extensive research has been done in this area, the behavior and fundamental principles are not yet fully understood [27,28,29] because, for example, such materials have extraordinary strength, but plasticity is too low, and also disks treated in this way have anisotropic properties between the center and the edges [29].

**Figure 1:**
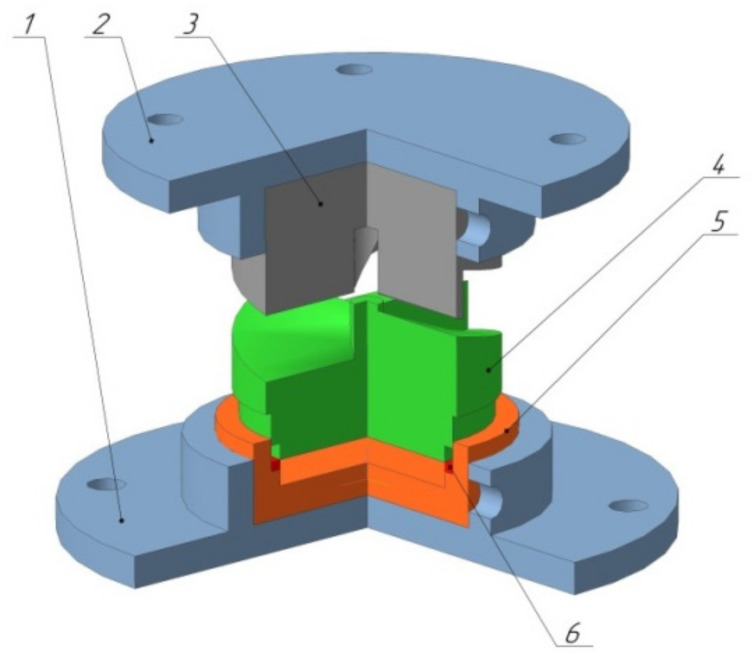
General view of the complete construction: 1—bottom carrier, 2—top carrier, 3—upper striker, 4—lower striker, 5—matrix, and 6—piston ring.

The geometric shape of the sample at HPT is designed so that the entire bulk of the material undergoes deformation under quasi-hydrostatic compression, which is ensured by the applied pressure of anvils and pressure from the outer layers of the sample. Due to this deformation scheme, the machined sample is not destroyed, even despite the high degree of applied deformation [30]. In spite of this, this method has a disadvantage in the radial inhomogeneity of the obtained sample, which, according to papers [24,31], can be reduced by increasing the number of revolutions. In 1979, S. Erbel in [32] suggested the HPT method to process ring-shaped workpieces, according to the proposed scheme. In practice, however, this technology is most often applied to disk-shaped workpieces [21,24,33]. Therefore, one of the stages of research was the development of a special die design that allows implementing this process of severe plastic deformation on ring workpieces. On the basis of modeling in the software package Deform, given in papers [34,35], design drawings were developed. The design consists of several parts: the upper striker which is driven by the progressive motion of the press; and the lower striker which is driven by the progressive motion of the upper striker and a matrix [34,35]. There are four periodic spiral-shaped indentations on the lower edge of the upper striker. There is a cylindrical hole in the center of the upper striker for the deforming element rod and for ensuring alignment of both strikers. The lower striker has several steps. This design solution is necessary because in this case we are talking about deformation of a ring workpiece, not a disk workpiece (Figure 1).

This design allows the high-pressure torsion process to be realized by a straight motion of the striker relative to the frame. When the press frame is lowered, the upper striker moves linearly with the press frame. A stationary lower striker is fixed on the bottom plate of the press. When the lower striker contacts the upper one, the upper striker transmits to the die a torque. As a result of the contact friction forces directed at an inclined angle to the responsive part of the die, the straight motion is transformed into a torsional motion. This results in the HPT of the piston ring.

The purpose of this work is to study the properties of antifriction rings obtained by severe plastic deformation by the HPT method in order to improve the service life of antifriction rings.

## 2. Materials and Methods

Rings of face seals are usually made of deformable brass of LZhMts59-1-1 grade (58.0-Cu; 40.0-Zn; 0.9-Fe; 0.7-Mn; 0.4-Al, weight %), so the laboratory experiment was carried out just on this material. The initial workpiece had a ring shape with a diameter of 55 mm, a width of 3 mm and a thickness of 3 mm. Brass grade LZhMts59-1-1 refers to manganese brass and has a high ductility and high strength. The alloy’s high ductility is due to the fine grain structure resulting from its alloying with iron. This brass is also highly corrosion resistant in seawater and atmospheric conditions, is quite ductile at high temperatures, so it withstands hot deformation well and is satisfactorily pressure treated at room temperature. It has good antifriction properties. Since this brass in the initial state has a two-phase structure α + β, which has low ductility, so in order to reduce the initial grain size, the ring workpieces were subjected to preliminary heat treatment. The preheating treatment consisted of annealing the brass at 600 °C and slow cooling together with the furnace.

**Figure 2:**
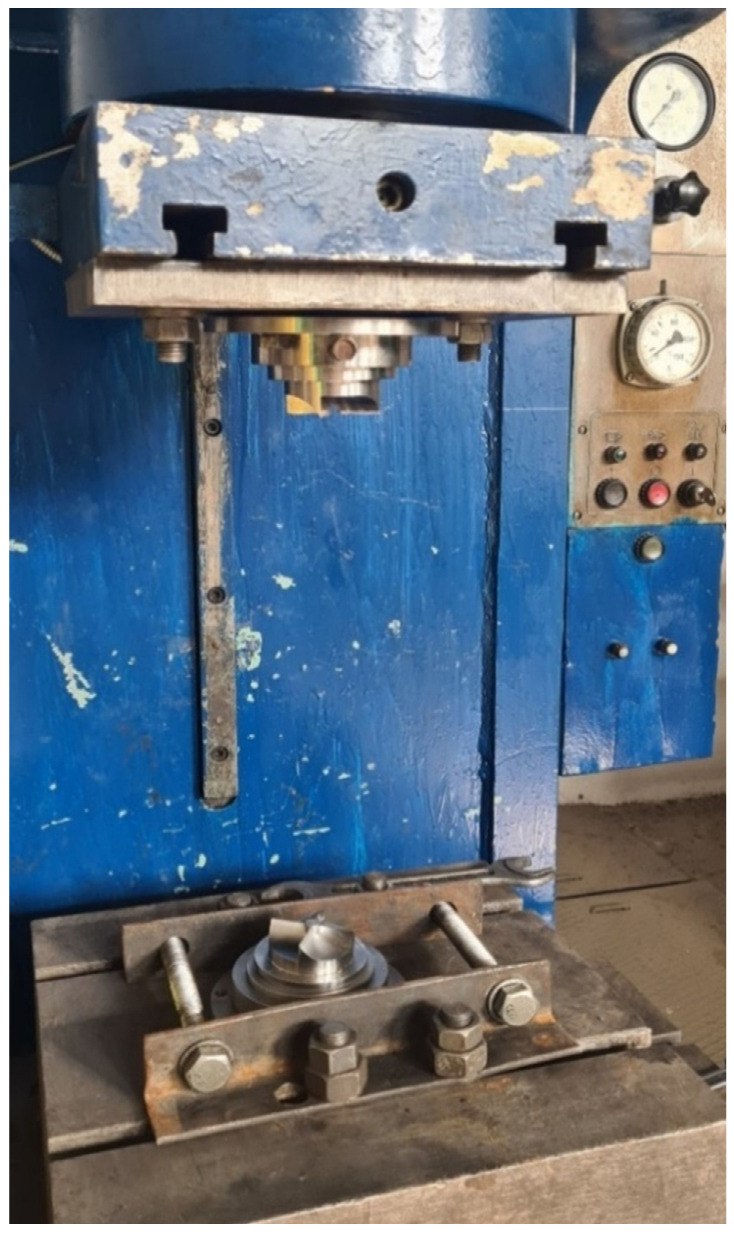
Structure assembly.

Experimental tooling for the HPT was made of 5XB2C steel on a CNC turn-milling machine (FS-300-05, LLC TD “Belarusian machines”, Smolensk, Belarus). The components of the structure were subjected to a special heat treatment after manufacturing to increase the strength properties. After all elements of the structure were manufactured, they were assembled. Thus, the lower holder was installed on the base plate of the hydraulic press and secured with tie bolts through the retaining angles (Figure 2). In the lower holder, the lower striker is installed, with a chamber for ring deformation, the upper matrix is installed from above on the sample.

**Figure 3:**
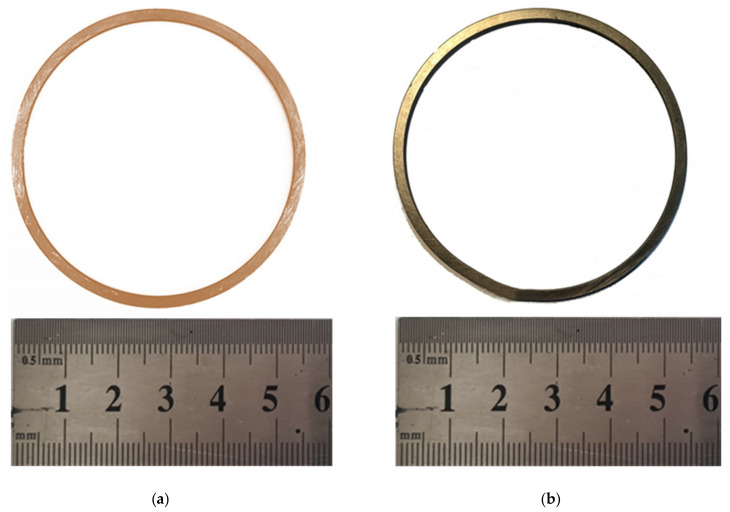
Type of workpieces: (**a**) initial state; and (**b**) after six deformation cycles by HPT method.

The structure was assembled and the experiment itself was carried out in the laboratory on a single-column hot-stamping crank press model PB 6330-02 (Almaty Heavy Machinery Plant, Almaty, Kazakhstan), the force of which is 1000 kN. Deformation was carried out at a temperature of 500 °C. Number of deformation cycles—6. The workpieces for deformation were heated in a Nabertherm resistance chamber furnace in a conventional (neutral) atmosphere. The heating temperature was set on the furnace panel and the holding time at the set temperature was set. After the end of heating operations, the workpiece was taken out of the furnace by pincers and fed into the working area of the die. The workpieces before and after deforming are shown in Figure 3.

Polished specimen for metallographic studies were prepared according to the standard method for this purpose. The rings were examined in the middle plane of the sample, as well as in two cross-sections: transverse and longitudinal, to avoid the influence of peripheral areas.

The fine structure was examined on a JEM2100 transmission electron microscope (TEM) (Jeol Ltd., Tokyo, Japan) at a magnification range of 1000 to 50,000 times. The TEM objects were prepared by polishing with a Tenupol-3 device at −28 °C and 20V. In order to have a more objective interpretation of the grain structure compared to TEM, the EBSD analysis was performed using a Philips XL-30 REM (MEMS and Nanotechnology Exchange, Arlington, VA, USA) with a field cathode. The accelerating voltage is 20 kV. To process the results, the laboratory software Tex SEM (4.2, 2018, Capterra, Arlington, VA, USA) was used. The misorientation was calculated between adjacent (contiguous) scanning points. The dimensions of the scanning stage were predetermined based on the measurement areas and the expected grain or sub-grain sizes. Scanning was performed on 50 × 50 μm^2^ slices in 0.2 μm increments. Different misorientations between grains were established using a minimum resolution of 2° misorientation. Due to the experimental accuracy of EBSD method, all low-angle boundaries with a misorientation of less than 2° were excluded from consideration. All scanned reference points with a confidence index of ~0.1 were excluded from the sets provided to improve the overall accuracy of the images. The colors of the grains on the map correspond to the orientations indicated in the stereographic triangle. Thus, different colors in adjacent grains correspond to a misorientation between these two grains of more than 2°. Grain boundaries are indicated either by white lines corresponding to misorientations at small angles of 2–15° or by black lines corresponding to misorientations at large angles >15°. The fraction of indexed diffraction patterns was 98% of the total number of measured points. On all EBSD cards, all points that were unindexed were removed during the standard replacement (cleaning) procedure. The surface of workpieces was prepared by jet polishing on a Tenupol-3 device [14].

**Figure 4:**
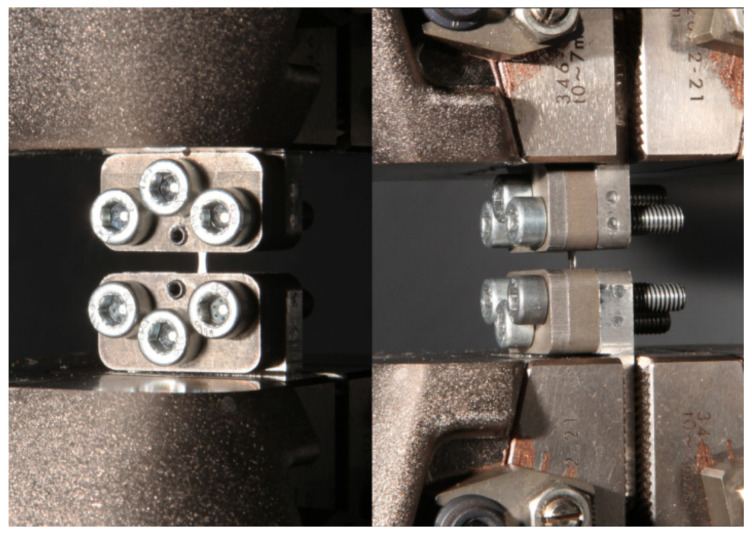
Stretching of the sample.

Mechanical tests for uniaxial tension were performed at ambient temperature on Instron 5882 machine (Instron, Norwood, MA, USA) with a strain rate of 1.0 mm/min (Figure 4). The tensile specimen were prepared according with method described in [32]. The strain of the sample was measured by an Instron strain gauge (Instron, Norwood, MA, USA). According to the results of tests, the strength and ductility characteristics were determined: yield strength, tensile strength, and elongation.

## 3. Results

**Figure 5:**
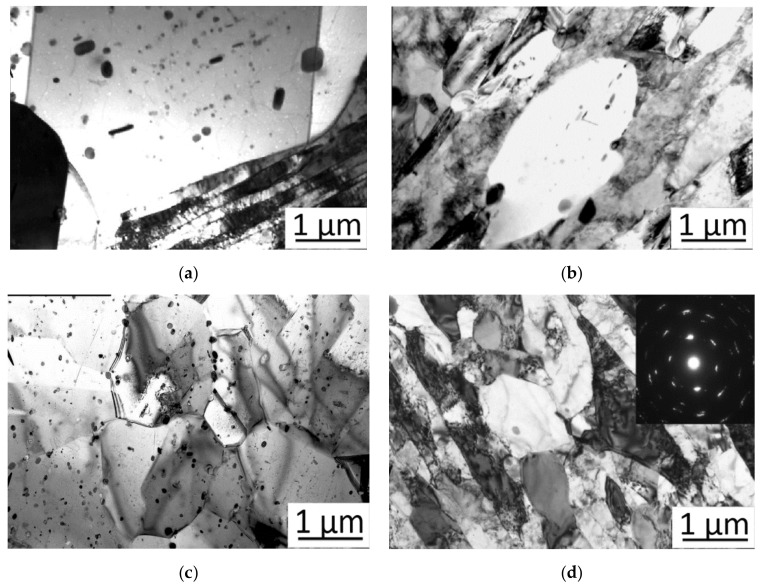
Microstructure of brass LZhMts59-1-1: (**a**) initial state; (**b**) after two deformation cycles; (**c**) after four deformation cycles; and (**d**) after six deformation cycles by HPT method.

The microstructure of brass in the initial state (after annealing) consists of α-phase (Figure 5a) where α-phase is a solid solution of zinc substitution in copper with a FCC lattice, which has high ductility, low strength, and hardness values. In the copper matrix there is a small amount of nanosized iron particles. As the number of deformation cycles increases, the β’-phase, an ordered solid solution based on CuZn intermetallic with a BCC lattice, begins to appear in the structure. This phase is characterized by higher hardness and brittleness than α-phase.

As can be seen from the microstructure during deformation dynamic softening processes occur (dynamic return, polygonization, and recrystallization), but they do not lead to complete softening of the deformed metal, as there is an excessive density of dislocations in the structure. And with increasing deformation cycles, the structure becomes less dispersed and more homogeneous.

**Figure 6:**
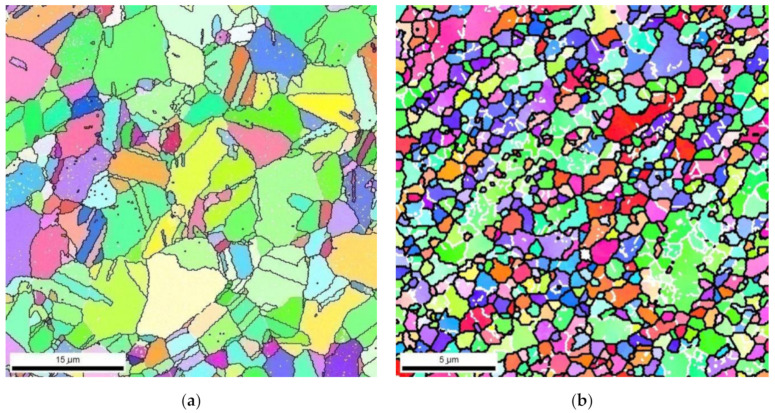
Microstructure orientation maps: (**a**) initial state; and (**b**) after six deformation cycles by HPT method.

To determine the crystal orientations and information about grain size, texture, and misorientation of the boundaries, EBSD analysis was performed. Orientation maps of brass LZhMts59-1-1 microstructure before and after 6 cycles of deformation by HPT method are shown in Figure 6.

**Figure 7:**
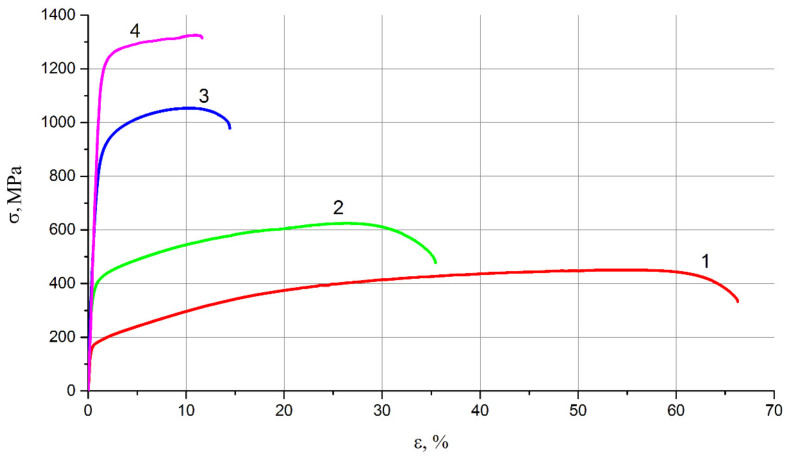
Tensile curves: (**1**) initial state; (**2**) after two deformation cycles; (**3**) after four deformation cycles; and (**4**) after six deformation cycles by HPT method.

After a detailed examination of microstructure evolution of brass rings, we see that severe plastic deformation by HPT method helps to significantly refine the microstructure, so the next stage of the study is to conduct mechanical tensile tests. Tensile tests determined the standard characteristics of strength and plastic properties: tensile strength (σ_B_), yield strength (σ_0.2_), percentage elongation (δ), and relative contraction (ψ). Tensile curves obtained as a result of tensioning of original samples and after two, four, and six cycles of deformation by HPT method are shown in Figure 7.

## 4. Discussion

Analysis of the microstructure after deformation showed that after the second cycle of deformation by HPT method, the microstructure of the copper matrix is refined and iron particles take a flat lens-like shape, with inclusions looking flatter and more elongated in the longitudinal section and lens-like in the transverse one. Grain size is refined from 17 μm in the initial state to 7 μm after the second cycle of deformation, the structure is multigrain (Figure 5b). The high deformation temperature led to dynamic softening, resulting in a grain that is not as intensely refined as it is when it is deformed at room temperature. After the fourth deformation cycle, the microstructure is refined to 3 μm, and “mottling” occurs in the structure, which is usually observed in strongly supersaturated solid solutions (Figure 5c). The shape of iron particles becomes flatter. Six cycles of deformation led to a refinement of the structure to 1 µm (Figure 5d), the structure is less dispersed and more homogeneous, the boundaries of the original grains are poorly traceable. A more detailed study of the type of arrangement of deformation bands demonstrated that first the nucleation of new grains proceeds near the initial high-angle boundaries, so there are recrystallized grains in the structure which arose after heating during deformation. As the number of deformation cycles increases up to six, the shape of iron particles becomes identical, both in transverse and longitudinal sections.

Both orientation maps shown in Figure 6 demonstrate a fairly homogeneous microstructure, but the distribution of boundaries by the angles of misorientation is very different. The average grain size of the original samples (after annealing) is ≈ 17 μm, and after six cycles of deformation by HPT method ≈ 1 μm. The fraction of the large-angle boundaries is 13% for the original samples and 79% after six cycles of deformation, which suggests the formation of ultrafine grained structure with the presence of a large number of large-angle boundaries. The portion of special boundaries revealed by EBSD analysis is minimal ~3%.

Based on TEM studies and EBSD analysis, it is clear that the type of structure obtained after six cycles of deformation by the HPT method is obtained by dynamic recrystallization.

The results of microhardness studies in both longitudinal and transverse sections showed a fairly homogeneous microhardness across the entire section. After six cycles of deformation the microhardness in comparison with an initial condition increases almost in three times, i.e., has increased from 820 MPa in an initial state up to 2115 MPa after deformation. Thus, the greatest growth of microhardness falls at the first two passes.

After annealing, the brass samples have an extended strain-hardening area (Figure 7, curve 1). As the number of deformation cycles increases, the strain-hardening area decreases. By analyzing the tensile diagrams, we can conclude that with an increase in the number of deformation cycles, the strength and yield strengths increase. Thus, the ultimate strength of the initial sample (after annealing), (Figure 7, curve 1), is 430 MPa, after two cycles of deformation (Figure 7, curve 2)—610 MPa, after four cycles of deformation (Figure 7, curve 3)—1050 MPa and after the sixth cycle (Figure 7, curve 4)—1295 MPa. The yield strength increases from 130 MPa in the initial state to 332 MPa, 805 MPa, and 1185 MPa, respectively. The plastic characteristics decrease with increasing number of deformation cycles, so the percentage elongation decreases from 65% in the initial state to 36% after the second cycle, to 15% after the fourth cycle and to 12% after the sixth cycle of deformation. This low percentage elongation shows that further deformation will make it absolutely brittle and not applicable in production without further heat treatment, so we do not increase the number of deformation cycles anymore.

Thus, according to the results of microstructural studies, it can be concluded that HPT in the new die is an effective method for grinding the microstructure and obtaining the finished workpieces. With an increase in the number of deformation cycles the microstructure becomes more developed and homogeneous, which favorably affects the mechanical properties. And the greatest dispersion of the structure, as well as an increase in mechanical characteristics occurs during the first two cycles of deformation.

## 5. Conclusions

Based on the results obtained from the conducted studies, the following main conclusions can be drawn, in particular, it was found that:

– the most intensive grain refinement in brass of LZhMts59-1-1 grade is observed during the first two cycles of deformation, and during the subsequent cycles the rate of dispersion of grains strongly decreases. Thus, six cycles of deformation allowed to disperse the structure from 17 μm to 1 μm;
– the fraction of large-angle boundaries is 13% for the original samples and 79% after six cycles of deformation, which suggests the formation of ultrafine-grained structure with the presence of a large number of large-angle boundaries without sharp texture. The portion of special boundaries revealed by EBSD analysis is minimal ~3%; and
– deforming brass rings by repeated HPT processing leads to significant hardening, and therefore will increase their service life.

## Data Availability

Data sharing is not applicable to this article.
